# Enhanced GIP Secretion in Obesity Is Associated with Biochemical Alteration and miRNA Contribution to the Development of Liver Steatosis

**DOI:** 10.3390/nu12020476

**Published:** 2020-02-13

**Authors:** Joanna Góralska, Urszula Raźny, Anna Polus, Agnieszka Dziewońska, Anna Gruca, Anna Zdzienicka, Aldona Dembińska-Kieć, Bogdan Solnica, Agnieszka Micek, Maria Kapusta, Krystyna Słowińska-Solnica, Małgorzata Malczewska-Malec

**Affiliations:** 1 Department of Clinical Biochemistry, Faculty of Medicine, Jagiellonian University Medical College, Kopernika Str. 15A, 31-501 Krakow, Poland; urszula.razny@uj.edu.pl (U.R.); a.polus@uj.edu.pl (A.P.); a.sliwa@uj.edu.pl (A.D.); a.gruca@uj.edu.pl (A.G.); anna.zdzienicka@uj.edu.pl (A.Z.); mbkiec@cyf-kr.edu.pl (A.D.-K.); mbsolnic@cyf-kr.edu.pl (B.S.); maria.kapusta@uj.edu.pl (M.K.); krystyna.slowinska-solnica@uj.edu.pl (K.S.-S.); m.malczewska-malec@uj.edu.pl (M.M.-M.); 2 Institute of Nursing and Midwifery, Faculty of Health Sciences, Jagiellonian University Medical College, Kopernika Str. 25, 31-501 Krakow, Poland; agnieszka.micek@uj.edu.pl

**Keywords:** GIP, obesity, miRNA, liver steatosis, FGF-21, FGF-19, cytokeratin-18, gut–liver cross-talk

## Abstract

Nutrient excess enhances glucose-dependent insulinotropic polypeptide (GIP) secretion, which may in turn contribute to the development of liver steatosis. We hypothesized that elevated GIP levels in obesity may affect markers of liver injury through microRNAs. The study involved 128 subjects (body mass index (BMI) 25–40). Fasting and postprandial GIP, glucose, insulin, and lipids, as well as fasting alanine aminotransferase (ALT), γ-glutamyltransferase (GGT), cytokeratin-18, fibroblast growth factor (FGF)-19, and FGF-21 were determined. TaqMan low density array was used for quantitative analysis of blood microRNAs. Fasting GIP was associated with ALT [β = 0.16 (confidence interval (CI): 0.01–0.32)], triglycerides [β = 0.21 (95% CI: 0.06–0.36], and FGF-21 [β = 0.20 (95%CI: 0.03–0.37)]; and postprandial GIP with GGT [β = 0.17 (95%CI: 0.03–0.32)]. The odds ratio for elevated fatty liver index (>73%) was 2.42 (95%CI: 1.02–5.72) for high GIP versus low GIP patients. The miRNAs profile related to a high GIP plasma level included upregulated miR-136-5p, miR-320a, miR-483-5p, miR-520d-5p, miR-520b, miR-30e-3p, and miR-571. Analysis of the interactions of these microRNAs with gene expression pathways suggests their potential contribution to the regulation of the activity of genes associated with insulin resistance, fatty acids metabolism, and adipocytokines signaling. Exaggerated fasting and postprandial secretion of GIP in obesity are associated with elevated liver damage markers as well as FGF-21 plasma levels. Differentially expressed microRNAs suggest additional, epigenetic factors contributing to the gut–liver cross-talk.

## 1. Introduction

Glucose-dependent insulinotropic polypeptide (GIP) is an intestinal hormone secreted from enteroendocrine K cells, with a broad range of physiological actions. As an incretin hormone, GIP is released within minutes upon intake of meal and, in the presence of elevated blood glucose, potentiates insulin secretion [1]. Several nutritional factors, including meal size, diet composition, glycemic index, or type of fat, can affect GIP secretion [2,3]. Chronic exposure to a high-fat diet in the presence of elevated blood glucose increases the expression of GIP in the gut [4], and induces K cell hyperplasia [5]. Mechanisms that lead to the secretion of incretin hormones are not fully understood, but involve taste receptors, G-protein coupled receptors (e.g., Fatty Acid Binding Protein 5 (FABP5)), sensing fatty acid derivatives, exposure to bile acids, and the microbiome [6,7]. Fat ingestion is also a strong stimulant of GIP release, but in the state of eu- or hypoglycemia, GIP does not influence insulin, but stimulates glucagon secretion, suggesting that GIP supports the maintenance of optimal glucose levels [8,9]. Reduced incretin effect is a feature of type 2 diabetes (T2D) [10]. GIP receptor down-regulation and desensitization have been suggested as potential causes of the diminished insulin response to GIP in T2D [11,12].

GIP also acts directly on adipocytes, immune cells, endothelial cells, bone cells, and neuronal cells [13]. Therefore, pleiotropic GIP actions involve the following: control of beta cell function and survival, promotion of nutrient storage and inhibition of lipolysis in adipose tissue, effect on inflammatory cytokines secretion, regulation of bone marrow cells’ differentiation, and regulation of bone metabolism [14,15,16,17]. Selected studies indicate that disturbed GIP signaling in obesity as well as in diabetes is associated with impairment of fat metabolism and liver fat accumulation, but the mechanisms are still poorly understood [3,18]. As the receptor for GIP (GIPR) is not found to be expressed on hepatocytes, the suggested relation of GIP with fatty liver disease may involve its direct and indirect influence on nutrients’ metabolism, as well as interactions with other endocrine factors regulating liver function.

Non-alcoholic fatty liver disease (NAFLD) refers to a spectrum of disorders ranging from simple hepatic steatosis to more severe manifestations, including non-alcoholic steatohepatitis (NASH), which can progress to fibrosis, cirrhosis, and liver failure. Subjects with NAFLD and NASH typically have elevated circulating concentrations of markers of liver injury, including alanine aminotransferase (ALT), aspartate aminotransferase (AST), and γ-glutamyltransferase (GGT) [19]. If biopsy or imaging of the liver are not possible, these markers of liver injury serve as easy, reliable surrogate measures of NAFLD [20]. For practical purposes, some algorithms (e.g., fatty liver index; FLI) were developed for the selection of subjects at greater risk for hepatic steatosis in the general population, based on routine measurements in clinical practice [21].

For diagnosis and staging of NASH, hepatocyte apoptosis markers, such as cytokeratin-18 (CK-18), can be useful [22]. Cytokeratin-18, being a major intermediate filament protein in hepatocytes, is cleaved by caspase 3 and released into the blood stream by hepatocytes undergoing apoptosis. The fragmented CK-18 (M30) serum level is associated with the presence of hepatic fibrosis. Cytokeratin-18 is an emerging risk factor and could be applied as a valuable index for non-invasive diagnosis of steatohepatitis, although it is not introduced into routine laboratory tests.

The discovery of endocrine fibroblast growth factors (FGF), including FGF-21 and FGF-19, has uncovered new mechanisms that regulate metabolism and lipid homeostasis, and has provided potential therapeutic targets for type 2 diabetes, obesity, and hepatic steatosis [23]. After extensive research over the past few years, hepatokine FGF-21 has emerged as a promising diagnostic marker for an accurate and non-invasive diagnosis of NAFLD [24,25]. FGF-21 is known to regulate energy homeostasis, glucose-lipid metabolism, and insulin sensitivity [26], though it is unclear which of these metabolic functions of FGF-21 underlie the association of plasma FGF-21 level and NAFLD. FGF-19 is produced and released from enteroendocrine cells and reaches the liver through the portal vein, where it interacts via activation of the FGFR4-klotho complex receptor [27]. The effects of FGF-19 in the liver involve suppression of de novo lipogenesis, increase of fatty acids oxidation, and suppression of bile acids synthesis; thereby, FGF-19 is thought to ameliorate hepatic steatosis and lipotoxicity. Activity of this enterokine underlies the gut–liver axis concept [28].

In most studies, the prevalence of NAFLD is higher in individuals with features of metabolic syndrome including obesity, diabetes, and hypertension. The relationship of body mass index (BMI), waist circumference, triglycerides, and metabolic syndrome with NAFLD is well documented [29]. However, there are limited data on GIP as a prognostic factor for NAFLD. A recent paper by Yamane & Harada [30] indicates an important role of GIPR signaling in adipose tissue in high fat diet -induced insulin resistance and hepatic steatosis in vivo [30]. The above-mentioned metabolic diseases can be mediated by circulating microRNAs (miRNAs), which constitute an important epigenetic mechanism for the regulation of gene expression. Extracellular miRNAs constitute promising, clinically-useful biomarkers for the NAFLD and hepatocellular carcinoma [31]. NAFLD development and progression is also modulated by miRNAs, which control at post-transcriptional level many complementary target mRNAs, and whose dysregulation has been shown to have predictive value in NAFLD, as reviewed by Dongiovanni et al. 2018 [32]. miRNAs are known to interact with products of different genes that regulate lipid synthesis, glucose and fatty acids utilization, inflammation, and apoptosis, which have been known to be epigenetically deregulated in NAFLD. Therefore, alteration of miRNA expression in response to nutrition and nutrition-related endocrine factors may contribute to liver steatosis. A recent study on the animal model underlies the role of miRNA, regulated by diet and insulin resistance, in cell-to-cell communication between hepatocytes and hepatic stellate cells, which may promote progressive NAFLD [33]. There are very little data on circulating miRNA in relation to plasma incretin levels, and it concerns mainly glucagon-like peptide-1 (GLP-1) not GIP [34]. Understanding how enhanced GIP secretion in obese humans can alter plasma miRNA profile and characterizing their target mRNA may help to elucidate potential biological mechanisms for contribution of elevated GIP to the development of liver steatosis.

In this study, the aim was to investigate whether exaggerated secretion of GIP in obesity may affect the markers of liver injury and liver targeted FGFs. We assessed the relationships of plasma levels of GIP to ALT, GGT, cytokeratin-18, and FLI, as well as FGF-19 and FGF-21. As GIP acts mainly after meal ingestion, we analyzed some risk factors for the development of fatty liver disease, not only fasting, but also in the postprandial state. In addition to biochemical markers, the study attempts to find miRNAs as molecular markers associated with high plasma GIP levels, which may contribute to the risk of fatty liver disease. Finally, we analyzed the roles of significantly regulated miRNAs in relation to their target genes. 

## 2. Materials and Methods 

### 2.1. Subjects

The study included 128 subjects with BMI exceeding the normal range (25–40 kg/m^2^), women and men, aged 25–65 years. All participants had no evidence of chronic diseases, particularly liver diseases, diabetes mellitus, endocrine disorders, kidney dysfunction, autoimmune disease, and inflammation. Exclusion criteria included therapy with hormones, anti-inflammatory, or other medicines/pharmaceuticals known to affect lipid or glucose metabolism; moderate to excessive use of alcohol; smoking; pregnancy; or lactation. Participants were drawn from the Bioclaims study (data from the study are deposited in Nutritional Phenotype Database http://www.dbnp.org/nutritional-instance/). The study was conducted in accordance with the Code of Ethics of the World Medical Association (Declaration of Helsinki) and with the Good Clinical Practice guidelines. The study protocol was approved by the Bioethics Committee of the Jagiellonian University, Krakow, Poland (file number: KBET/82/B/2009 and 1072.6120.57.2017), and all individuals gave oral and written informed consent prior to inclusion in the study.

### 2.2. Anthropometry Measurements

Body weight, height, waist, and hip circumferences and blood pressure were measured. Body composition was estimated with the eight-polar bio-electrical impedance analysis using the Segmental Body Composition Analyzer TANITA BC 418 MA (Tanita Corporation, Tokyo, Japan)**.** Ethanol intake was validated by analysis of a seven-day diary of food intake.

### 2.3. Sample Collection and Analysis

Venous blood samples were collected through an intravenous catheter fasting and during an oral glucose tolerance test and meal tolerance test into K-EDTA tubes (Sarstedt AG & Co. Nümbrecht, Germany). Plasma was separated from blood cells within 30 min by centrifugation (10 min at 3000× *g*, at 4 °C). For biochemical tests (except free fatty acids (FFAs)), plasma samples were immediately stored at −80 °C until analyzed. For microRNA analysis, venous blood was collected into PAXgene Blood RNA Tubes (Becton Dickinson, Bedford, NJ, USA) and stored at −80 °C until analyzed.

### 2.4. Oral Glucose Tolerance Test (OGTT) 

An oral glucose tolerance test was performed according to *World Health Organization/International Diabetes Federation* guidelines. Tests were performed during morning hours (08:00–11:00) after a 10 h overnight fast. Blood samples were collected before glucose load (fasting) and then 30, 60, 90, and 120 min after the ingestion of 75 g of glucose dissolved in 250 mL of water.

### 2.5. Meal Tolerance Test (MTT)

The day before the test, participants ate a last low-fat meal before 6 p.m. (two slices of bread without any fatty products, and unsweetened tea). Test meals were given at 07:30 and postprandial studies were performed from 07:30 to 13:30. The test meal contained light bread—50 g, butter—20 g, cream cheese—60 g, pork loin roast—100 g, and mayonnaise—40 g. This meal contained 73% fat, 16% protein, and 11% carbohydrates, with a caloric value of 1018 kcal (the composition of MTT was described in detail previously [35]). Venous blood samples were collected before the meal (fasting) and postprandially 2, 4, 6, and 8 h after the test meal.

### 2.6. Biochemical Tests

All biochemical tests were performed in fasting plasma samples. Glucose, insulin, and GIP were measured additionally in blood samples collected during the OGTT and GIP, triglycerides, and free fatty acids (FFAs) were also measured in blood samples collected during MTT.

Free fatty acids concentrations were measured immediately in fresh plasma by an enzymatic quantitative colorimetric method (Roche Diagnostics GmbH, Berlin, Germany). Plasma glucose, total cholesterol, HDL-cholesterol, and triglycerides were assayed by automated, standard enzymatic colorimetric methods using the MaxMat Analyzer (MaxMat SA, Montpellier, France). LDL-cholesterol was calculated using the Friedewald formula. Serum insulin was determined by the immunoradiometric method (DIAsource Immunoassys, Louvain-la-Neuve, Belgium) and read using the gamma counter (LKB Instruments, Victoria, Australia). Homeostasis model assessment of insulin resistance (HOMA-IR index) was calculated as a ratio (fasting glucose (mmol L^−1^) × fasting insulin (µU mL^−1^)]/22.5). To measure plasma GIP concentrations, the ELISA kit (Human GIP [Total] 96-Well Plate Assay (EMD Millipore, St Charles, MO, USA)) was used. The limit detection of the GIP assay used was 8.2 pg/mL. Activities of ALT and GGT were assayed in a clinical laboratory by the standard biochemistry method using an automated analyzer. The fatty liver index (FLI) is based on scoring algorithm involving BMI, waist circumference, triglycerides, and GGT, and was calculated according to Bedogni et al. (2006).


FLI = (e ^0.953*loge (triglycerides) + 0.139*BMI + 0.718*loge (GGT) + 0.053*waist circumference − 15.745^)/(1 + e ^0.953*loge (triglycerides) + 0.139*BMI + 0.718*loge (GGT) + 0.053*waist circumference - 15.745^) * 100

Cytokeratin-18 concentrations were determined by the M30 Apoptosense ELISA (PEVIVA, VIVALAVIDA, Sundbyberg, Sveden), which measures the levels of soluble caspase-cleaved keratin 18 (CK-18) fragments containing the K18Asp396 neo-epitope. The assays for FGF-19 and FGF-21 employed the quantitative ELISAs (Human FGF-19 Quantikine ELISA, and Human FGF-21 Quantikine ELISA, respectively, R&D Systems Inc. Minneapolis, MN, USA). The limit of detection of human FGF-19 and FGF-21 immunoassays was 1.17 pg/mL and 4.67 pg/mL, within-run coefficient of variation (CV) was 4.83% and 3.43%, and between-run CV was 5.13% and 7.5%, respectively.

### 2.7. Isolation and Real-Time PCR of miRNA 

Total RNA, including miRNA, was isolated from plasma, using the GeneMATRIX Universal RNA Purification Kit (EURx, Gdańsk, Poland) and RNA quality was assessed in an Agilent Bioanalyzer 2100 using the RNA 6000 Nano kit (Agilent Technologies, Santa Clara, CA, USA).

Relative miRNA expression was determined in 18 samples from subjects representing the high GIP group (*n* = 9) and low GIP group (*n* = 9). Representative subjects drawn from both groups were similar according to age, sex (77% female), and BMI, while they reflected the same differences between the two groups they represented (fasting and postprandial GIP levels and liver injury markers). For relative quantification of miRNA, TaqMan low density array (TLDA) was used (Thermo Fisher Scientific, Waltham, MA, USA). Reverse transcription was performed with TaqMan MicroRNA Reverse Transcription Kit components and MegaPlex Human Pool A and B RT primers. The preamplification step was enabled by Megaplex™ PreAmp Primers and then TaqMan^®^ Array Human MicroRNA A+B Cards Set v3.0 were employed for the accurate quantitation of 754 human microRNA by real-time PCR. The arrays were run on the 7900HT Fast Real-Time PCR system (ThermoFisher, Waltham, MA, USA). According to recommendations [36], a miRNA was considered non-informative if C_T_ values were >35 in >80% of samples. Relative miRNA levels were expressed as fold of change (fold of change (RQ) = geometric mean 2^(−ΔCt high GIP)^ / geometric mean 2^(−ΔCt lowGIP)^), where U6 snRNA was used as endogenous control.

The known miRNA gene targets were extracted using the mirPath v.3 DIANA TOOLS. The miRNA–gene interaction network was generated through Cytoscape with ClueGO [37].

### 2.8. Statistical Analyses

Data distribution was assessed by Shapiro–Wilk test, which indicated that data were usually not normally distributed. Homogeneity of variance was confirmed by Levene’s test. For anthropometric and biochemical parameters, data are presented as the median and lower and upper quartile (Q2 (Q1–Q3)). The two-sided Mann–Whitney U test was applied for comparison of these variables between two groups with low or high GIP. Data distributions, power of tests, confidence intervals, and other statistical calculations were performed using the Statistica software (StatSoft, v.13 PL). Logistic regression analysis was used to evaluate the contributions of high plasma GIP to the risk for elevated liver markers. Odds ratios (ORs) and 95% confidence intervals (95% CIs) for high GIP were calculated. Linear regression analysis was used to evaluate the association between fasting plasma GIP, GIP response to MTT, GIP response to OGTT, and fatty liver risk factors. Variance inflation factors (VIFs) were presented to quantify how much the standard errors of the estimated coefficients are inflated by the existence of correlation among the predictor variables. Areas under curves (AUCs) were calculated with the trapezoidal rule. The relative expression levels of miRNA were reported as the means and standard deviations. Two-sample, two-tailed Student’s t-test comparing the 2^(–ΔCt)^ values of the two groups was performed for *p* value calculation (DataAssist Software v.3.01). *p* < 0.05 was considered statistically significant and differentially expressed miRNA were used for signaling pathways analysis by Cytoscape (v.3.4.0).

## 3. Results

Associations of plasma fasting and postprandial GIP levels with factors that may influence NAFLD were investigated in this study. For this purpose, we analyzed liver injury markers (ALT, GGT, CK-18), liver targeted fibroblast growth factors (FGF-19, FGF-21), as well as circulating miRNA profile in relation to plasma GIP concentrations.

### 3.1. Baseline Characteristics of Subjects

The baseline characteristics of the subjects are summarized in Table 1.

All subjects (95 women and 33 men) included in the study were obese or overweight, the median BMI was 32.28 (Q1–Q3: 29.84–34.77) kg/m^2^, the median body fat was 38.30% (Q1–Q3: 33.50%–42.00%). Fasting plasma levels of GIP ranged from 5.36 pg/mL to 144.97 pg/mL, with a median of 26.16 pg/mL (Q1–Q3: 18.29–38.54). Secretion of GIP in response to glucose (OGTT) or meal (MTT) challenge measured as AUC values was also very diverse within this cohort (Table 1). ALT activity in the majority of subjects remained within the normal range (<40 IU/L), and only 5/128 subjects (4%) had higher plasma ALT activity. Median GGT activity was 18 IU and 85% of subjects had GGT <40 IU/L. The median of cytokeratin-18 fragments levels was 115 (Q1; Q3: 86–159). In serum from 28/128 subjects (22%), CK-18 levels were above 180 U/L, although in 90% of all subjects CK-18 values did not exceed 296 U/L. Fatty liver index (FLI) values above 60, recognized as predictive for liver steatosis, were found in 71/128 subjects (55%) from our cohort.

### 3.2. Association of Plasma GIP with Fatty Liver Risk Markers

In the whole cohort of obese subjects, we observed a correlation of fasting GIP with ALT (Spearman correlation coefficient *r* = 0.22, *p* = 0.015) and with FLI (Spearman correlation coefficient *r* = 0.27, *p* = 0.002). To examine in detail the association of plasma GIP with fatty liver risk markers, linear regression models were tested (Table 2).

This analysis revealed that the fasting plasma GIP level was significantly associated with ALT (independently of sex and age—model 1), with fasting triglycerides, and with FGF-21 (independently of sex, age, BMI, and fasting glucose—model 2). The standardized β-coefficient indicates that the increase of fasting GIP by 22 pg/mL is associated with the increase of FGF-21 by 34 pg/mL. An enhanced GIP response to high-fat meal intake (GIP AUC MTT) was found as a factor independently influencing not only fasting, but also postprandial triglycerides levels and, interestingly, higher GGT activity in both models. GIP response to glucose oral intake (GIP AUC OGTT) was also related to fasting triglycerides, which provide consistent data for the association of circulating GIP with triglycerides (Table 2). In all regression models, VIF was low (VIF < 2), indicating that there was no multicolinearity.

In separate logistic regression models adjusted for age and sex, subjects in the upper tertile of GIP were at significantly increased risk for elevated ALT compared with those in the lower tertiles: ALT > 23 IU/L OR 3.16 (95% CI 1.23–8.13); ALT > 31 IU/L, OR 4.82 (95% CI 1.46–15.91) (Table 3).

After further adjustment for BMI and fasting glucose, the associations of fasting plasma GIP with elevated ALT remained significant: ALT > 23 IU/L OR 3.11 (95% CI 1.13–8.57); ALT > 31 IU/L, OR 4.31 (95% CI 1.20–15.43). FGF-21 values were dichotomized using 187 pg/mL (median) or 254 pg/mL (3rd tertile) as cut-off points for logistic regression analysis. The high plasma GIP (>3rd tertile) was also an independent factor for increased risk (2.46 (1.05–5.84)) of high plasma FGF-21 (>3rd tertile) (Table 3). Compared with the participants in the two lower tertiles of GIP, the adjusted (age, sex) OR for elevated FLI (>73%) was 2.42 (95% CI 1.02–5.72) for those in the upper tertile of GIP. Moreover, OR for significantly elevated FLI (>87%) was 1.04 (95% CI 1.01–1.07) with the increase of fasting GIP by every 1 pg/mL. This association was adjusted for age, sex, BMI, and fasting plasma glucose levels. The results showed that fasting plasma GIP levels predict elevated liver markers—ALT, FLI, and FGF-21.

### 3.3. Fatty Liver Risk Markers in High GIP Subjects

On the basis of the fasting plasma GIP level, two groups were selected from among the patients with excess body weight. The cut-off point was set at 34 pg/mL, which states the 66th percentile of plasma GIP concentration in this cohort (Table 1). These two groups (high GIP versus low GIP) did not differ in terms of age, BMI, and body fat content. However, the subjects with higher plasma GIP had increased waist circumference and waist-to-hip ratio (WHR), indicating visceral adiposity. Even concerning the difference in gender distribution in both groups, the trend was also observed in the women subgroup, which constitutes the majority of subjects. The tendency for abdominal obesity in women with high GIP was expressed as greater waist circumference (Q2 (Q3–Q1): 100 (95–110) versus 96 (91–103), *p* = 0.052) and WHR (Q2 (Q3–Q1): 0.87 (0.84–0.91) versus 0.83 (0.80–0.89), *p* = 0.051). High GIP group was characterized by elevated fasting glucose, with impaired fasting glucose (5.6–6.9 mmol/L) in half of the cases, and enhanced insulin resistance HOMA-IR (Q2 (Q3–Q1): 3.68 (2.72–5.42) versus 2.70 (2.13–4.33), *p* = 0.021). Although fasting plasma total cholesterol, LDL-cholesterol, and FFA were comparable in both groups, subjects with high plasma GIP showed an unfavorable lipid profile regarding elevated triglycerides (Q2 (Q3–Q1): 1.57 (1.17–2.28) versus 1.16 (0.9–1.49), *p* = 0.002) and decreased HDL-cholesterol (Q2 (Q3–Q1): 1.32 (1.16–1.5) versus 1.20 (1.11–1.32), *p* = 0.004), compared with those with low GIP levels. 

GIP release in response to a high-fat meal as well as in response to oral glucose load (reflected by GIP AUC OGTT and GIP AUC MTT) was significantly greater in the high GIP group compared with the low GIP group (Table 1). Elevated postprandial GIP levels were accompanied by increased glucose and insulin levels in response to the meal, as shown by glucose AUC MTT (Q2 (Q3–Q1): 9.89 (9.46–10.44) versus 9.28 (8.86–9.92), *p* = 0.001) and insulin AUC MTT (Q2 (Q3–Q1): 43.10 (32.69–63.48) versus 34.68 (25.55–51.83), *p* = 0.028). Obese subjects characterized by high GIP levels also showed greater triglycerides response to a high-fat meal, reflected by significantly higher TG values, at every time point after meal ingestion during MMT: 2 h (Q2 (Q3–Q1): 2.31 (1.47–3.08) versus 1.9 (1.45–2.46), *p* = 0.048); 4 h (Q2 (Q3–Q1): 2.60 (1.90–3.76) versus 2.07 (1.40–3.00), *p* = 0.032); 6 h (Q2 (Q3–Q1): 2.13 (1.20–2.82) versus 1.44 (1.05–2.30), *p* = 0.015); 8 h (Q2 (Q–Q1): 1.57 (0.97–2.33) versus 1.09 (0.79–1.53), *p* = 0.006). The above results point to the disturbed postprandial glucose and lipid metabolism in high GIP obese patients.

**Figure 1:**
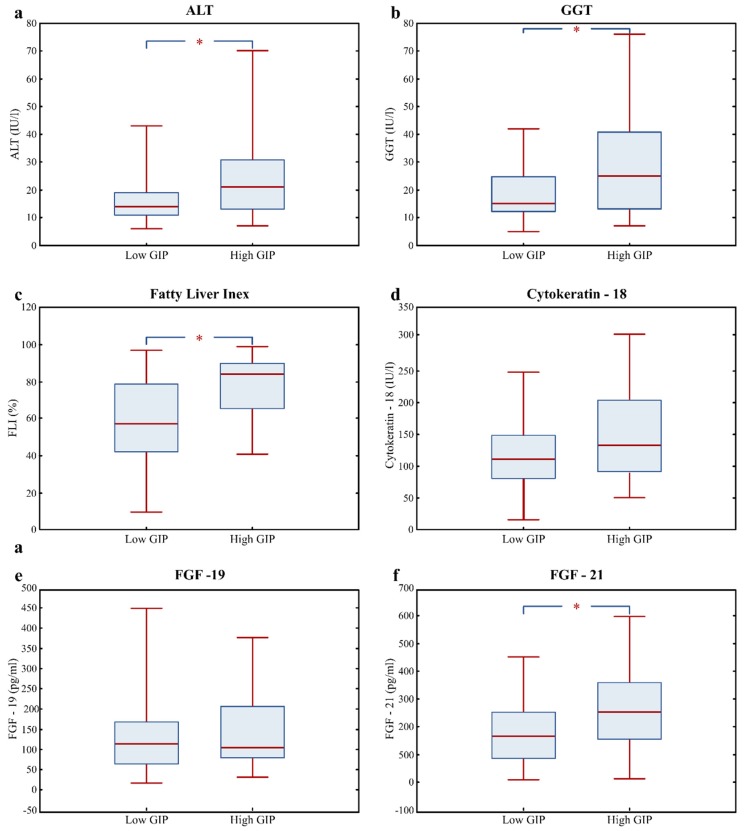
Fatty liver risk markers: ALT (**a**), GGT (**b**), FLI (**c**), cytokeratin-18 (**d**), FGF-19 (**e**), and FGF-21 (**f**) in the Low GIP (*n* = 85) and High GIP (*n* = 43) groups of patients. Values are presented as median (line), upper and lower quartile (box), and minimum and maximum (whiskers). Significant differences are indicated as * *p* < 0.05 (Mann–Whitney U test, Statistica softaware v.13). Abbreviations: High GIP—group of subjects with fasting plasma GIP level >66th percentile, Low GIP—group of subjects with plasma fasting GIP level ≤66th, ALT—alanine aminotransferase, GGT—gamma-glutamyltransferase, FGF-19—fibroblast growth factor-19, FGF-21—fibroblast growth factor-21.

Alanine aminotransferase, GGT, and cytokeratin-18 fragments were determined as biochemical markers of liver injury and the fatty liver index was calculated. The percentage of subjects with FLI >60 in the high GIP group was 74%, whereas in the low GIP group, it was 46% (*p* < 0.05). The plasma activity of both liver enzymes ALT and GGT, as well as calculated FLI, was significantly elevated in subjects with GIP ≥66th percentile (Table 1, Figure 1a–c). In this group, only a tendency for elevated CK-18, a marker of liver cells apoptosis, was observed (Q2 (Q3–Q1): 132.5 (91.1–193.2) versus 110.8 (80.5–148.3, *p* = 0.090), compared with the low GIP group (Figure 1d). However, plasma CK-18 >180 IU was found in 15 subjects from the low GIP group and 13 subjects from the high GIP group.

Two liver targeted FGFs, enterokine FGF-19 and hepatokine FGF-21, were also analyzed in the groups of obese subjects according to plasma fasting GIP levels. There was no difference in the concentration of FGF-19 between these groups (Figure 1e). In contrast, the levels of hepatokine FGF-21 differ between both groups, being significantly higher in the high GIP group (Q2 (Q3–Q1): 253 (154–357) versus 165 (85–255), *p* = 0.007) compared with the low GIP group (Figure 1f).

### 3.4. MicroRNA profile in High GIP Subjects

**Figure 2:**
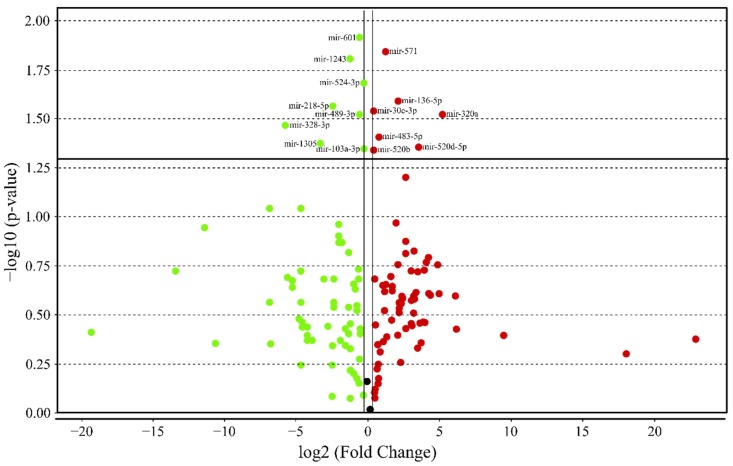
Volcano plot shows the relative expression of microRNAs in the high GIP versus low GIP group; fold change boundary: 1.2, *p*-value boundary: 0.05 (prepared using DataAssist Software v.3.01).

Analysis of microRNA relative expression revealed the miRNA profile related to high GIP plasma level in obesity. For 754 microRNAs tested with TLDA in each blood sample, 174 types of miRNAs were detected, and 15 miRNAs were differentially expressed in analyzed groups, including 7 up regulated miRNAs and 8 downregulated miRNAs (Figure 2).

**Figure 3:**
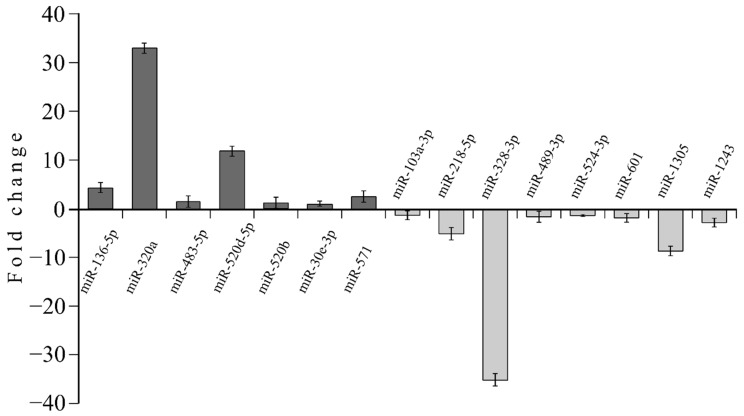
Quantitative PCR (TaqMan low density array (TLDA)) showing the expression of microRNAs in the high GIP versus low GIP group. Relative miRNA levels are expressed as fold of change, with U6 snRNA used as endogenous control; upregulated miRNA—black bars, downregulated miRNA—gray bars (*n* = 18; *p* < 0.05; t-test, DataAssist Software v.3.01)**.**

Relatively increased miRNAs’ expression in the high GIP patients concerns miR-136-5p, miR-320a, miR-483-5p, miR-520d-5p, miR-520b, miR-30e-3p, and miR-571; as well as miR-103a-3p, miR-218-5p, miR-328-3p, miR-489-3p, miR-524-3p, miR-601, miR-1305, and miR-1243 for relatively decreased miRNAs’ expression, compared with low GIP patients (Figure 3).

**Figure 4:**
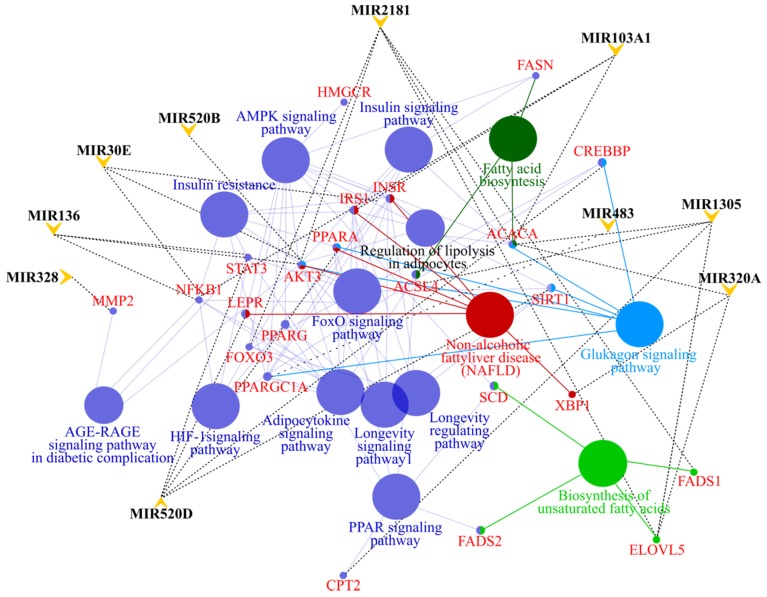
Interactions of microRNAs with gene pathways. The known miRNA gene targets were extracted using the mirPath v.3 DIANA TOOLS. The miRNA-gene interaction network was generated through Cytoscape with ClueGO.

Analysis of the interactions of differentially expressed microRNAs with gene expression pathways suggests the potential contribution of selected miRNAs in the regulation of the activity of genes associated with insulin resistance, fatty acids metabolism, and adipocytokine (TNF-α, leptin) signaling (Figure 4).

Significantly upregulated miR-320a as well as miR-520b and miR-520d-5p have the potential to inhibit the expression of genes involved in insulin signaling such as *PIK3CA*, *AKT3*, *SHC4*, *SOS1*, *KRAS*, and *MAPK1*. MicroRNA-320a together with upregulated miR136-5p may inhibit *PPARGC1*, which is a crucial transcription factor regulating energy homeostasis, fatty acids oxidation, and mitochondrial biogenesis. Downregulated miR-103a-3p and miR-1305 may influence lipogenesis by target genes *FASN* and *ACCA*, respectively. Additionally, the deficit of miR-1305 may also result in insufficient inhibition of *ACSL6*, leading to intensive synthesis of ceramides, and thus linking excess nutrients and inflammatory cytokines (e.g., tumor necrosis factor-α, TNF-α) to the induction of insulin resistance. In the high GIP group, leptin signaling may be disturbed by the interaction of miR-520d with the *LEPR* gene transcript, and further by other interactions, including the following: miR-320a with *JAK_2_* and miR-520b with *STAT_3_*. Another important target gene of miR-520d, abundant in subjects with high plasma GIP levels, is *PPARA*. Deficiency of this nuclear receptor leads to disturbed fatty acids metabolism and promotes HFD-induced hepatic lipids accumulation and steatohepatitis.

## 4. Discussion

In recent years, much attention has been paid to studying the role of incretin hormones in regulating metabolism, especially in the context of diabetes and obesity treatment, with more emphasis on GLP-1. However, it seems that GIP is of significant importance in the development of obesity and its complications, through interactions with various body organs. The hypothesis was raised that GIP signaling participates in the development of hepatic steatosis [30]. Thus, our study aimed to investigate whether enhanced secretion of GIP in obese humans may affect markers of liver injury, liver targeted FGFs, and plasma miRNA profile.

In the presented study, fasting plasma GIP values varied widely among studied 128 subjects with excess body weight, correlated well with postprandial GIP secretion, and were dichotomized using 34 pg/mL (3rd tertile) as cut-off points to select “high GIP” and “low GIP” subjects. The above results highlight the disturbed postprandial glucose and triglycerides metabolism in high GIP obese patients. Subjects with low GIP, despite obesity, seemed to be insulin sensitive and normolipemic. Under physiological conditions, diet-induced GIP action is beneficial for the physiology of adipose tissue promoting the nutrient uptake in response to nutrient excess. GIP directly induces energy accumulation in adipocytes by increasing lipoprotein lipase activity, stimulation of lipogenesis, as well as by enhancing plasma membrane GLUT4 expression and glucose uptake. Indirectly, GIP potentiates the anabolic action of insulin, which in total allows appropriate storage of nutrients and protects against lipotoxicity [38]. However, several studies reported elevated GIP levels in obese humans compared with lean humans [39]. Recently, GIP (3-30) NH2, a naturally occurring peptide, was shown to block the GIPR in humans and decrease GIP-induced insulin secretion as well as adipose tissue blood flow and TG uptake [40]. Killion et al. [41] provided a preclinical validation of a therapeutic approach to treat obesity with anti-GIPR antibodies. However, there is no agreement on whether GIP receptor agonism or antagonism lowers body weight [42]. Recently published in The Lancet, data from a phase II trial on dual human agonist with an imbalance in favour of GIP agonism showed significantly better efficacy with regard to weight loss than did selective stimulation of GLP-1 receptor [43,44]. Several studies have highlighted the direct relationship between overnutrition, increased levels of GIP, and the development of obesity and its selected metabolic consequences including diabetes [17]. In human plasma, GIP levels are increased with obesity and correlate with body mass index [39], but the exact role of GIP in the development of obesity and diabetes is not fully understood and various concepts are suggested. Some observations indicate that hyperglycemia directly downregulates GIP receptors in beta cells, disturbing late-stage insulin release [45]. Increased postprandial GIP secretion positively correlates with insulin resistance, and chronic local and systemic inflammation, which may involve IL-6 signaling [46]. The obesity-associated inflammatory state plays a key role in the development of the hepatic steatosis. 

The present study demonstrated that the plasma activity of both liver enzymes ALT and GGT as well as calculated FLI, but not CK-18, were significantly elevated in subjects with high plasma GIP levels. Martin-Rodriguez et al. (2017) showed a correlation of ALT with liver fat content and increased insulin resistance, suggesting that ALT, even when within the normal range, may be useful for NAFLD categorization when evaluating NAFLD’s systemic relationships. This study shows that optimal serum ALT cut-off to predict NAFLD is 23 IU/L [47], although there is no general consensus on the upper reference limit of ALT (40 IU/l is the most common in medical practice). A study on obese patients showed that elevated ALT and GGT levels correlated significantly with the incidence of steatohepatitis and fibrosis [48]. Fatty liver index (FLI) was developed as a simple and accurate predictor of hepatic steatosis in the general population of north Italy, and further, it has been validated on several European populations [21]. The large multicentre study showed that FLI (like ALT), discriminated between patients with and without steatosis with an area under ROC (AUROC) of 0.79 (inter-quartile range (IQR) = 0.74, 0.84), accurately matched the observed percentages of patients with hepatic steatosis, although it could not quantitatively predict fatty liver disease [49]. The usefulness of CK-18 to differentiate between steatohepatitis and simple steatosis has been reported in many studies [22,50,51]. CK-18 is a major component of intermediate filaments of hepatocytes, and the circulating fragment of CK-18 can specifically reflect the degree of hepatocellular apoptosis, which is a characteristic of NASH. Thus, cytokeratin-18 (M30) is a biomarker of disease severity in NAFLD, indicating progression to steatohepatitis [22]. In our study, increased ALT, GGT, and FLI associated with normal CK-18 (M30) suggest elevated risk for simple steatosis, but not steatohepatitis in high GIP subjects, without symptoms of advanced hepatocytes apoptosis.

In a previous study on diabetic patients, higher fasting GIP levels were also related to higher fasting and postprandial triglyceride levels and ALT [52]. The pathophysiology of NAFLD includes (apart from dietary fat contribution) proinflammatory cytokines, hepatic and adipose tissue insulin resistance, lipotoxicity, and oxidative stress. A reduced hepatic glucagon resistance, together with an impaired incretin effect, may be additional mechanisms [53]. Although the functional receptor for GIP (GIPR) was not found in the liver, this incretin hormone may play a role in hepatic steatosis exerting pleiotropic effects in other tissues. The group of Musso et al. [3] showed convincing results, indicating that GIP response to saturated fatty acids (SFA) ingestion is prolonged in non-diabetic patients with NASH and is correlated with liver disease, an unfavorable dynamic adipokine profile, and β cell dysfunction, which provides a rationale for GIP antagonism in these subjects [3]. The results reported by Junker showed that non-diabetic patients with NAFLD have normal secretion of GIP and GLP-1, but a reduced incretin effect, although patients with cirrhosis have elevated concentrations of GIP and GLP-1, and a reduced incretin effect [54,55]. Recent papers underlie an important role of GIPR signaling in adipose tissue in HFD-induced insulin resistance and hepatic steatosis in vivo with no direct effect on fat accumulation [30]. It was shown that deletion of GIPR signaling in adipocytes results in the decrease in interleukin (IL)-6 production in adipose tissue and subsequent decrease in fat synthesis in the liver through the IL-6–SOCS3–SREBP-1c pathway. Insulin resistance, liver weight, hepatic steatosis, and body weight, but not fat volume, were reduced in HFD-fed GIPRadipo-/- mice [56]. Data from another study show that GIP response to glucose absorption may play a role in sucrose induced liver fat accumulation by regulating the expression of Socs2 [57]. Moreover, central GIP related pathways are suggested to be involved in epigenetic programming of peripheral gene expression related to a reduction of fatty acids oxidation, observed as increased methylation of promoter of carnitine palmitoyltransferase gene (CPT1α) [58]. GIP acts in the brain by increasing the expression of the orexigenic hypothalamic neuropeptide Y (NPY) and by modulating central AKT-mechanistic target of rapamycin (mTOR) signaling, resulting in decreased fat oxidation in muscle and fatty liver [2].

In our study, among two liver targeted FGFs analyzed—FGF-19 and FGF-21—only FGF-21 was found significantly associated with high circulating GIP. Meta-analysis of several studies provide clues about the role of FGF-21 as a key regulator of hepatic lipid metabolism in humans and suggest that serum FGF-21 can be used as a biomarker for NAFLD, being the most promising in combination with other markers, such as CK-18 [24]. FGF-21 has multiple metabolic actions in animal models of obesity that include enhancing insulin sensitivity, decreasing triglyceride concentrations, and causing weight loss. In lean rodents, FGF-21 expression is strongly induced in liver by prolonged fasting through peroxisome proliferator-activated receptor gamma coactivator protein-1alpha (PGC-1alpha). FGF-21, in turn, induces PGC-1alpha and stimulates hepatic gluconeogenesis, fatty acid oxidation, and ketogenesis, playing a key role in coordinating the adaptive starvation response [59]. Expression of FGF-21 is also upregulated postprandially through peroxisome proliferator-activated receptor gamma in white adipose tissue [60]. FGF-21 is known to increase metabolic flux and to reduce hepatic steatosis, but the mechanisms responsible for these effects are not fully discovered. The recent study on PGC-1alpha-deficient mice revealed that, although FGF-21 effects such as stimulation of physical activity or energy expenditure depend on PGC-1alpha, the enhanced hepatic oxidative capacity is mediated by other transcription factors [61]. The genetic variability of FGF-21 was found to be potentially associated with macronutrient consumption, and risk of obesity and type 2 diabetes in humans [62]. Interestingly, in adipocytes, FGF-21 altered the expression of 18 secretory genes and enhanced by 1.15-fold the release of dipeptidyl peptidase IV (DPP IV)—the enzyme involved in GIP (and GLP-1) inactivation [63]. This report raises the hypothesis that GIP secreted in response to meal ingestion, in the presence of high plasma FGF-21 concentration is quickly cleaved by DPP IV and does not act properly. This relationship, together with results from our study, emphasize the importance of an efficient gut–adipose tissue–liver axis.

Fibroblast growth factor 19 functions mainly as an entero-hepatic signal to regulate bile acids homeostasis (through inhibition of CYP7A1), and its deficiency impairs liver regeneration in mice [64,65]. The FGF-19 has the ability to maintain glucose homeostasis through inhibition of gluconeogenesis, in the mechanism involving inhibition of the cAMP response element binding protein and PGC1-α signaling cascade [66]. Conversely, administration or overexpression of FGF-19 in mice provided protection from diet-induced obesity as a result of increased hepatic fatty acid oxidation *via* suppression of acetyl-CoA carboxylase 2 and stearoyl-CoA desaturase 1 [67,68]. Furthermore, the circulating FGF-19 concentration is reduced in patients with NAFLD, suggesting that dysregulated FGF-19 expression might contribute to the pathogenesis of NAFLD [28,69]. Recently, in a randomized, placebo-controlled trial, an FGF-19 analogue, NGM282, was shown to rapidly improve the liver fat content, independently of weight loss and BMI, over 12 weeks of treatment [70]. Significant improvements in ALT, AST, and non-invasive serum fibrosis biomarkers including pro-peptide of type III collagen and the total enhanced liver fibrosis score were also noticed [70]. FGF-19 decreases bile acids secretion, including the ursodeoxycholic acid, which was recently proved to increase GLP-1, but not GIP, secretion [71]. Thus FGF-19, though associated with nutrient metabolism, GLP-1 response, and insulin sensitivity, seems not to be associated with plasma GIP levels, as confirmed in our study. The metabolic functions of endocrine FGFs can be regulated by miRNAs. Downregulation of miR-34a increases the levels of the FGF-21 receptor components, resulting in FGF21/SIRT1-dependent induction of genes inducing browning of adipose tissue and improved hepatic FGF-21 signaling and lipid oxidation [72].

In our study, besides the analysis of the association between plasma GIP levels and liver markers, we attempted to identify molecular mechanisms leading to liver damage in patients with impaired GIP signaling. For this purpose, we compared the plasma microRNA profile in obese patients with high versus low plasma GIP levels. Analysis of the interactions of differentially expressed microRNAs with gene expression pathways suggests the potential contribution of selected miRNAs to the regulation of the activity of genes associated with insulin resistance, fatty acids metabolism, and adipocytokine (TNF-α, leptin) signaling. Upregulated miRNAs in the high GIP group included miR-136, miR-320a, miR-483, miR-520d, miR-520b, miR-30e-3p, and miR-571.

Enhanced inflammatory cytokines expression and apoptosis were induced by microRNA-136 through negative regulation of Klotho expression in HK-2 cells [73]. As Klotho inhibits NF-κB and Wnt signaling pathways, over-expression of miR-136 could reverse this inhibition [74]. Other results indicate that miR-136 acts by targeting NF-κB signaling pathway to promote apoptosis in cervical carcinoma [75]. Thus, over-expression of miRNA-136 may also be responsible for the upregulated NF-κB pathway found to be related to high plasma GIP levels [46]. Moreover, liver receptor homolog-1 (LRH-1) was identified as a direct target gene of miR-136 [75]. In the liver, LRH-1 was established as a critical regulator of reverse cholesterol transport [76] as well as a crucial regulator of bile acid homeostasis [77]. Thus, in high GIP subjects, the miR-136 over-expression may lead to dyslipidemia and liver steatosis through suppression of LRH-1 expression.

We identified strongly upregulated miR-320a in high GIP versus low GIP subjects. Transcriptomic data from mice pancreatic tissue point to miR-320 as the negative regulator of the mRNA associated with PI3K-Akt pathway, and hence disturbed nutrient metabolism and diabetes [78]. Recently, it has been documented that miR-320 inhibits SIRT1 expression via targeting the 3′ UTR region of FoxM1 [79]. SIRT1 is the key sensor of metabolic states, which, in response to intracellular NAD+ levels, directly deacetylates and regulates transcription factors, such as PGC1-α, PPARs, FOXO1, LXR, RXR, and SREBPs, important for nutrient metabolism. The role of adequate SIRT1 levels for proper liver function is well documented, as SIRT1 deletion results in hepatic steatosis and inflammation, and SIRT1 activators improve hepatosteatosis and insulin resistance [80,81]. MicroRNA-320 may also regulate the development of autophagy by targeting hypoxia-inducible factor-1α (HIF-1α) and mTOR under hypoxic conditions [82]. Dysregulation of the mTOR pathway—a key controller of lipid metabolism, regulating lipogenesis in the liver, lipolysis in white adipose tissue, and adipogenesis—may promote liver steatosis and development of NAFLD [83].

We found miR-571 to be upregulated in the high GIP group. Previously, miR-571 has been described as being associated with type 2 diabetes as well as significantly correlated with microvascular complications of diabetes [84]. Moreover, alterations of serum miR-571 levels in patients with chronic liver disease and its up-regulation in human hepatocytes and hepatic stellate cells in response to the pro-fibrogenic cytokine TGF-β reflected their putative roles as a mediator of fibrogenic and inflammatory processes in distinct cellular compartments involved in the pathogenesis of liver cirrhosis [85].

In our study, miR-520d-5p was upregulated in high GIP patients. A luciferase reporter assay revealed binding sites for miR-520d-5 on TEA domain transcription factor 1 (TEAD1) target gene [86]. It has been proven recently that knockdown of TEAD1 decreases the expression of PGC1α and suppresses mitochondrial biogenesis, glycolysis, and oxygen consumption in endothelial cells [87]. Reducing the number of active mitochondria and slowing down the rate of mitochondrial oxidation as a result of down-regulation of TEAD1 and PGC1-α may contribute to disturbed fatty acid catabolism, and consequently to the development of fatty liver in high GIP patients.

The present study revealed significantly lower miR-328 expression in high GIP patients compared with the low GIP group. In the experimental model of osteosarcoma cells, the inverse relationship between miR-328 and MMP-2 expression was found and inhibition of miR-328 resulted in the enhanced production of MMP-2 [88]. Upregulation of miR-328 repressed the expression of TGF-β2 and extracellular matrix (ECM) proteins and prevented renal fibrogenesis [89]. Enhanced production of matrix metalloproteinases, including MMP-2, and ECM proteins may lead to tissue remodeling, as well as in the liver. A recent study indicates that miR-328-3p targets GLUT1, leading to a significantly lower glucose uptake and decline in intracellular levels of glucose and lactate [90]. Consequently, a significant miR-328-3p deficiency may increase nutrient uptake, essential for liver steatosis.

Previous studies on mice or rat models showed other microRNAs, including miRNA-27a, miR-29, miR-122, miR-132, miR-150, and miR-181b, to be involved in regulating hepatic lipids’ metabolism and to be associated with liver steatohepatitis [91,92,93,94]. Other reports indicate miR-34a, miR-335, miR-33a/b, miR-143, and miR-103/107 as those that target liver, as reviewed by Rottiers et al. [95] and Dongiovanni et al. [32]. The above types of microRNAs, as well as microRNAs characteristic for obesity, were not identified in our study as differentially expressed in high GIP subjects. The most likely reasons are that all of our studied subjects were comparable according to BMI and body fat content, as well as the fact that they did not have a clinically diagnosed liver disease.

The incretin hormones can modulate the expression of miRNAs, although most of the data concern GLP-1 not GIP. GLP-1 was found to upregulate miR-758, miR-27a, miR-192, miR-212, and miR-132 and downregulate miR-23 and miR-375 [34]. Both up and down regulation result in a reduction of blood glucose by improving beta-cell function and increasing insulin secretion. Little is known about regulation of miRNA expression by GIP, though there is a report on overexpression of miR-375 stimulated also by GIP [96]. The authors suggest that this circulating miRNA may originate from enteroendocrine cells or the brain, and could exert a diverse effect on lipid and glucose metabolism in the liver. Thus, circulating levels of miRNA may respond to signaling molecules released by the gut, but further experimental studies are warranted.

The main limitation of our study is the lack of liver imaging data, which would allow us to assess the level of liver steatosis in obese patients. The multiparametric capability of ultrasonography and MR imaging obviously gives high diagnostic performance towards liver injury, and these tests should be taken into account when planning further studies on GIP as a prognostic factor for NAFLD. However, our objective was to elucidate how high circulating GIP may influence factors contributing to the development of fatty liver disease. Thus, we focused on the gut–liver axis, especially liver-targeted FGFs and microRNAs as potent mechanisms of the inter-organ crosstalk. Another limitation of our study is the small number of samples available for miRNA profiling. Nevertheless, we made an attempt to carry out miRNA analysis in subjects representative for both high GIP and low GIP groups, in accordance with most current guidelines [36]. For this reason, we suggest that miRNAs differentially expressed, according to circulating GIP, in our study, should be validated on a larger group of subjects. Circulating miRNAs originate from death cells or from microvesicles that are actively released from cells. Future studies should also analyze the distribution of miRNAs in the different compartments, protein versus vesicles, that could provide additional information regarding the identification of a specific miRNA pattern associated with disturbed GIP signaling. 

## 5. Conclusions

Overall, in the current study, we indicate that exaggerated fasting and postprandial GIP secretion in obesity is associated with elevated ALT, GGT, FLI, as well as FGF-21 plasma levels. Differentially expressed microRNAs suggest additional, epigenetic factors, contributing to the development of liver steatosis in patients with impaired GIP signaling. Understanding how high plasma GIP levels influence liver may help to elucidate potentially biological mechanisms for determining treatment strategies and prognosis.

## Figures and Tables

**Table 1:** Characteristics of subjects included in the study, comparison of groups according to fasting plasma glucose-dependent insulinotropic polypeptide (GIP) level.

	All (*n* = 128)	Low GIP (*n* = 85)	High GIP (*n* = 43)	
	Median (Q1; Q3)	Median (Q1; Q3)	Median (Q1; Q3)	*p* *
**Sex (% of female)**	**74%**	**81%**	**61%**	**0.021 ^§^**
**Age (yrs)**	47 (39; 57)	46 (39; 57)	54 (41.5; 58)	0.134
**BMI (kg/m^2^)**	32.28 (29.84; 34.77)	32.03 (28.87; 34.55)	32.97 (31.13; 36.02)	0.065
**Waist circumference (cm)**	100 (94; 110)	99 (93; 108)	110 (97; 117)	**0.009**
**Women**	98 (93; 106)	96 (91; 103)	100 (95; 110)	0.052
**Men**	114 (106; 118)	114 (107; 116)	116 (105; 121)	0.505
**WHR**	0.88 (0.82; 0.96)	0.85 (0.81; 0.93)	0.91 (0.84; 0.98)	**0.016**
**Women**	0.84 (0.81; 0.90)	0.83 (0.8; 0.89)	0.87 (0.84; 0.91)	0.051
**Men**	0.99 (0.89; 1.51)	1.01 (0.98; 1.04)	0.98 (0.96; 1.03)	0.263
**Body fat (%)**	38.30 (33.50; 42.00)	38.1 (33.58; 42.05)	38.8 (32.6; 42)	0.959
**Systolic blood pressure (mmHg)**	130 (120; 140)	125 (120; 134.5)	130 (120; 140)	0.184
**Diastolic blood pressure (mmHg)**	84 (80; 90)	82 (80; 90)	90 (80; 90)	0.111
**Fasting glucose (mmol/l)**	5.1 (4.8; 5.6)	5.0 (4.8; 5.4)	5.6 (5.0; 6.0)	**<0.001**
**Fasting insulin (µIU/mL)**	13.05 (9.68; 19.03)	12.4 (9.10; 18.00)	15.00 (11.40; 20.55)	0.075
**HOMA-IR**	2.93 (2.17; 4,56)	2.7 (2.13; 4.33)	3.68 (2.72; 5.42)	**0.021**
**GIP (pg/mL)**	26.2 (18.3; 38.5)	19.44 (15.27; 26.12)	44.86 (38.72; 53.43)	**<0.001**
GIP AUC MTT (ng/mL*min)	347.7 (279.4; 436.9)	320.3 (248.8; 389.7)	401.0 (324.7; 573.6)	**<0.001**
GIP AUC OGTT (ng/mL*min)	63.8 (47.6; 86.1)	60.97 (46.91; 79.73)	74.42 (58.18; 94.43)	**<0.013**
**FFA (mmol/l)**	0.67 (0.52; 0.86)	0.67 (0.52; 0.83)	0.66 (0.53; 0.93)	0.714
FFA AUC MTT (mol/l*min)	1.45 (1.22; 1.78)	1.41 (1.22; 1.68)	1.56 (1.17; 2.03)	0.338
**TG (mmol/l)**	1.24 (0.92; 1.80)	1.16 (0.90; 1.49)	1.57 (1.17; 2.28)	**0.002**
TG AUC MTT (mol/l*min)	3.47 (2.43; 4.89)	3.22 (2.30; 4.34)	4.19 (2.83; 5.84)	**0.017**
**Cholesterol total (mmol/l)**	5.43 (4.82; 6.07)	5.39 (4.8; 6.03)	5.46 (4.94; 6.3)	0.311
**HDL-cholesterol (mmol/l)**	1.28 (1.13; 1.48)	1.32 (1.16; 1.5)	1.20 (1.11; 1.32)	**0.004**
**LDL-cholesterol (mmol/l)**	3.45 (2.87; 4.13)	3.41 (2.90; 4.08)	3.54 (2.78; 4.39)	0.527
**ALT (IU/l)**	16.0 (12.0; 22.0)	14.0 (11.0; 19.0)	21.0 (13.5; 30.5)	**0.004**
**GGT (IU/l)**	18.0 (12.0; 31.0)	15.0 (12.0; 25.0)	24.0 (15.3; 40.5)	**0.007**
**Fatty Liver Index (%)**	70.5 (45.1; 86.1)	57.4 (42.0; 79.1)	84.2 (66.4; 90.3)	**0.000**
**Cytokeratin; 18 (U/l)**	115.4 (86.1; 159.5)	110.8 (80.5; 148.3)	132.5 (91.1; 193.2)	0.090
**FGF-19 (pg/mL)**	108.8 (65.5; 178.4)	112.6 (62.8; 168.4)	104.2 (81.0; 206.2)	0.567
**FGF-21 (pg/mL)**	187.4 (120.2; 285.5)	165.3 (85.3; 255.4)	253.3 (154.3; 357.0)	**0.007**

* Except the first row, the significance of all differences were tested using the Mann–Whitney U test; ^§^ the significance of differences in the percentage of female was tested using the Chi-square frequency test; values are median (lower quartile; upper quartile), except sex values expressed as percentage of female, (*n* = 128). Abbreviations: High GIP—group of subjects with fasting plasma GIP level >66th percentile, Low GIP—group of subjects with plasma fasting GIP level ≤66th, BMI—body mass index, WHR—waist-to-hip ratio, HOMA-IR—homeostatic model assessment, GIP—glucose-dependent insulinotropic polypeptide, AUC—area under curve, MTT—meal tolerance test, OGTT—oral glucose tolerance test, FFA—free fatty acids, TG—triglycerides, ALT—alanine aminotransferase, GGT—gamma-glutamyltransferase, FGF-19—fibroblast growth factor-19, FGF-21—fibroblast growth factor-21.

**Table 2:** Linear regression models of the association between fasting and postprandial GIP plasma level and fatty liver risk factors.

	Model 1 β (95% CI)	Model 2 β (95% CI)
**Fasting triglycerides**		
Fasting GIP	**0.21 (0.06–0.36)**	**0.20 (0.04–0.35)**
*R2*	18%	19%
*VIFs*	<1.0	<1.3
GIP AUC MTT	**0.21 (0.05–0.37)**	**0.20 (0.04–0.36)**
*R2*	21%	24%
*VIFs*	<1.0	<1.2
GIP AUC OGTT	**0.16 (0.01–0.31)**	**0.17 (0.02–0.33)**
*R2*	16%	18%
*VIFs*	<1.0	<1.2
**TG AUC MTT**		
GIP AUC MTT	**0.24 (0.09–0.40)**	**0.23 (0.07–0.39)**
*R2*	24%	25%
*VIFs*	<1.0	<1.2
**ALT**		
Fasting GIP	**0.16 (0.01–0.32)**	0.14 (−0.02–0.3)
*R2*	21%	24%
*VIFs*	<1.0	<1.3
**GGT**		
GIP AUC MTT	**0.17 (0.03–0.32)**	**0.15 (0.01–0.30)**
*R2*	35%	36%
*VIFs*	<1.0	<1.2
**FGF-21**		
Fasting GIP	**0.20 (0.03–0.37)**	**0.22 (0.05–0.4)**
*R2*	7%	10%
*VIFs*	<1.1	<1.3
GIP AUC MTT	1.14 (−0.04–0.31)	1.17 (−0.01–0.34)
*R2*	5%	7%
*VIFs*	<1.1	<1.3

Model 1 adjusted for sex and age (*n* = 128); model 2 adjusted for sex, age, BMI, and fasting glucose (*n*=128); R^2^—coefficient of determination expressed in %; VIF—variance inflation factor. Abbreviations: GIP AUC MTT—area under curve of GIP concentration during meal tolerance test, GIP AUC OGTT—area under curve of GIP concentration during oral glucose tolerance test, TG AUC MTT—area under curve of serum triglycerides concentration during meal tolerance test, ALT—alanine aminotransferase, GGT—gamma-glutamyltransferase.

**Table 3:** Contributions of high plasma GIP level (odds ratios and 95% confidence interval (CI)) to the risk of elevated liver markers—logistic regression analysis.

High GIP	OR (95% CI) for ALT > 23 IU/l	OR (95% CI) for ALT > 31 IU/l	OR (95% CI) for FLI > 73%	OR (95% CI) for FGF-21 > Median	OR (95% CI) for FGF-21 > 3rd Tertile
Model 1 *(adjusted for age and sex)*	3.16 (1.23–8.13) *	4.82 (1.46–15.91) *	2.42 (1.02–5.72) *	2.43 (1.12–5.40) *	2.53 (1.14–5.67) *
Model 2 *(adjusted for age, sex, BMI, fasting glucose)*	3.11 (1.13–8.57) *	4.31 (1.20–15.43) *	1.85 (0.57–6.06)	2.04 (0.89–4.75)	2.46 (1.05–5.84) *

Odds ratios (OR) and 95% CI (confidence interval) here indicate changes associated with upper tertile of GIP; * *p* < 0.05 (*n* = 128); Abbreviations: High GIP—group of subjects with fasting plasma GIP level >66th percentile; ALT—alanine aminotransferase, FLI—fatty liver index, BMI—body mass index.
